# Editorial: Advances in phage applications: deciphering phage biological and ecological mechanisms through metagenomics

**DOI:** 10.3389/fmicb.2026.1822387

**Published:** 2026-03-30

**Authors:** Tasha M. Santiago-Rodriguez, Gary A. Toranzos

**Affiliations:** 1 Department of Molecular Virology and Microbiology, Baylor College of Medicine, Houston, TX, United States; 2 Department of Biology, Universidad de Puerto Rico, San Juan, Puerto Rico

**Keywords:** bioinformatics, metagenomics, phage, phageome, virus

Bacterial and archaeal viruses are the silent and lamentably underestimated architects of the biological world, acting as primary drivers of microbial evolution, ecosystem stability, genetic diversity and overall evolution. As we navigate an era defined by rapid leaps in metagenomics, artificial intelligence, and structural biology, bacteriophage research is also undergoing profound changes. This Research Topic, “*Advances in phage applications: deciphering phage biological and ecological mechanisms through metagenomics*,” curates 10 original research and review articles that collectively expand our understanding of phage diversity and evolution, host interactions, ecological roles, and their therapeutic potential. We invite the reader to peruse these publications, as they present some enticing new data on these usually overlooked biological entities in spite of their overall importance.

## Unraveling the viral “dark matter” through metagenomics

A central theme of this Research Topic is the use of high-throughput sequencing to expand our knowledge on complex ecosystems. Wei et al. provide a landmark analysis of the porcine gut microbiome, identifying over 10,000 prophage genomes. Their work reveals the pig intestine as a massive reservoir of lysogenic novelty, where prophages actively influence host fitness by encoding auxiliary metabolic genes, antibiotic resistance determinants, and virulence factors. Together, these findings expand viral reference databases and emphasize the ecological and functional importance of prophages in gut microbial communities. Aside from the data presented, the reader may also see the potential danger for possible future outbreaks of human and animal disease as a result of the mismanagement of porcine waste.

Expanding this lens to a global scale, Wei and Chen (a) and Wei and Chen (b) introduce a staggering catalog of over 740,000 phage genomes across diverse habitats. Their findings demonstrate that nearly one-third of global phage species are absent from current reference databases. By mapping CRISPR-based interaction networks, they uncover the ecological rules governing phage communities and identify novel molecular tools, such as a phage-encoded Cas12 nuclease, hidden within these viral lineages.

Complementing these large-scale surveys, Lu et al. provide a focused ecological discovery in the marine environment. By isolating two novel phages infecting bacteria from the genus *Erythrobacter*, they shed light on the viral predators of aerobic anoxygenic photoheterotrophs, key players in the ocean's carbon cycle, that represent a new genus within the *Casjensviridae*.

## Deciphering bacteria-phage arms race

As metagenomics provides a catalog of the virosphere, other contributors to this issue focus on the mechanics of how these parts function. The structural nuances of the “arms race” between phages and bacteria are explored by Matusiak et al., who use AlphaFold2-Multimer modeling to re-examine the tail spike architecture of *Caudoviricetes*. Their work suggests that we have systematically underestimated the complexity of receptor-binding proteins (RBPs), revealing small, “hand-like” accessory proteins that may be the true mediators of host specificity. The described mechanisms may shed light on other types of virus-host interactions as well.

The genomic signatures of this conflict are synthesized in a comprehensive review by Zhang et al., who frame the interaction as a coevolutionary struggle shaped by an ever-evolving repertoire of restriction systems, CRISPR-Cas immunity, and viral anti-defense innovations. Understanding these mechanisms is a prerequisite for effective clinical intervention using bacteriophages in the short-, medium and long-term interactions.

## From computational prediction to clinical applications

The transition from environmental discovery to clinical application is bridged by advances in machine learning and comparative genomics. Chen et al. introduce MoEPH, a framework that integrates protein language model embeddings to predict phage-host interactions with unprecedented accuracy. This tool offers a scalable, transparent solution for host-range inference, moving the field closer to rational, data-driven phage therapy.

Complementing advances in host-range prediction, Lixi et al. introduce StackPVP, a stacked ensemble framework designed to enhance the identification of phage virion proteins (PVPs). Moving beyond conventional sequence-derived descriptors, the authors harness evolutionary information encoded in position-specific scoring matrices and integrate multiple feature representations within a two-layer ensemble architecture. By combining diverse base classifiers with a random forest meta-classifier and rigorous feature selection strategies, StackPVP demonstrates robust performance across established benchmark datasets. As viral genome sequencing continues, tools such as StackPVP may help transform raw sequence data into biologically meaningful insights.

In a direct application of these genomic principles to the clinical crisis of multidrug resistance, Pang et al. analyze 250 *Acinetobacter* phage genomes. By resolving these into 12 proteomic clusters, they identified a high-potential lineage with exceptional host breadth. Their identification of conserved endolysins and HNH endonucleases provides a toolkit for the next generation of phage-derived antimicrobials.

Finally, the methodology and history of the field are given their due. Panteleev et al. provide a methodological roadmap, bridging century-old classical plaque assays with modern single-cell imaging, microfluidics, and Hi-C technologies. This review serves as an essential guide for researchers navigating the multi-scale complexity of modern virology.

Collectively, these 10 contributions illustrate that the modern study of bacteriophages is an inherently interdisciplinary endeavor. By integrating ecological discovery with mechanistic precision and computational power, this Research Topic provides a foundation for the next generation of phage biology. As the Research Topic editors we truly thank the authors and reviewers for their visionary contributions and hope this Research Topic inspires continued efforts to unlock the vast biological and applied potential of the virosphere.

